# Using action observation to study superior motor performance: a pilot fMRI study

**DOI:** 10.3389/fnhum.2013.00819

**Published:** 2013-11-27

**Authors:** Carl-Johan Olsson, Peter Lundström

**Affiliations:** ^1^Ageing and Living Conditions Programme, Centre for Population Studies, Umeå UniversityUmeå, Sweden; ^2^Umeå centre for Functional Brain Imaging, Umeå UniversityUmeå, Sweden

**Keywords:** motor representations, action observation, fMRI, expert performance, cognitive neuroscience

## Abstract

The most efficient way to acquire motor skills may be through physical practice. Nevertheless, it has also been shown that action observation may improve motor performance. The aim of the present pilot study was to examine a potential action observation paradigm used to (1) capture the superior performance of expert athletes and (2) capture the underlying neural mechanisms of successful action observation in relation to task experience. We used functional magnetic resonance imaging to measure regional blood flow while presenting videos of a hockey player shooting a puck toward a hockey goal. The videos (a total of 120) where stopped at different time frames with different amount of information provided, creating a paradigm with three different levels of difficulty to decide the fate of a shot. Since this was only a pilot study, we first tested the paradigm behaviorally on six elite expert hockey players, five intermediate players, and six non-hockey playing controls. The results showed that expert hockey players were significantly (*p* < 0.05) more accurate on deciding the fate of the action compared to the others. Thus, it appears as if the paradigm can capture superior performance of expert athletes (aim 1). We then tested three of the hockey players and three of the controls on the same paradigm in the MRI scanner to investigate the underlying neural mechanisms of successful action anticipation. The imaging results showed that when expert hockey players observed and correctly anticipated situations, they recruited motor and temporal regions of the brain. Novices, on the other hand, relied on visual regions during observation and prefrontal regions during action decision. Thus, the results from the imaging data suggest that different networks of the brain are recruited depending on task experience (aim 2). In conclusion, depending on the level of motor skill of the observer, when correctly anticipating actions different neural systems will be recruited.

## INTRODUCTION

The most efficient way to acquire motor skills may be through extensive motor training. Motor performance via motor skill training relies on the creation of internal motor representations, which enable us to repeat and, thereby, strengthen learned motor skills and improve performance (Dushanova and Donoghue, 2010). The motor representation comprises the entire movement, including the plan for the movement as well as the intended result (Kandel et al., 2000). Moreover, the motor representation is suggested to precede the execution, and could, therefore, be detached from the actual execution and exist on its own (Jeannerod, 2006). Interestingly, during action observation it has been suggested that the same neurons as are used during action performance are activated, which is referred to as the mirror neuron system (Rizolatti and Craighero, 2004). Further, it has also been shown that action observation may be used to enhance motor performance (Mattar and Gribble, 2005). Thus, if observing a movement also recruits the motor representation, then the representation itself may be strengthened, which may lead to performance improvements. In a related field to action observation, motor imagery, accessing the motor representation is also central. In this research field, studies have shown that task specific physical experience is needed in order to recruit motor regions of the brain during motor imagery, without such experience visual and pre-frontal regions of the brain will be recruited (see Olsson et al., 2008; Olsson and Nyberg, 2010, 2011). Similar suggestions have been reached within the observation literature. For example Calvo-Merino et al. (2005) showed that professional dancers could only recruit the mirror neuron regions of the brain when watching dance moves within their own motor repertoire. When observing dance like moves, other regions were recruited. Moreover, Aglioti et al. (2008) showed that expert basketball players used body cues to predict the fate of a basketball shot before the ball left the hand, which was also associated with a greater neural response in motor regions. Basketball coaches that no longer performed on expert level had to rely of the trajectory of the ball with less motor activity. In studies of action anticipation temporal occlusion paradigms have successfully been used to study points at which experts pick up the most information. For example, studies of anticipatory skills in badminton showed how experts are superior compared to novices in anticipating the landing position of strokes, which required fine tuned mechanisms in order to pick up information from the player’s body kinematics early in the decision process (Abernethy and Zawi, 2007). Moreover, performance on temporal occlusion tasks are associated with expertise in the relevant sport, an advantage that is unchanged even if the stimulus material are changed from video clips to point-light information (Abernethy and Zawi, 2007). Further, the differences between experts and novices are even larger for the early occluded clips (e.g., Jackson et al., 2006). Thus, it is now widely recognized that experts pick up relevant information earlier than novices (see e.g., Abernethy et al., 2008). However, the neural underpinnings of such behavior are not completely understood. Wright et al. (2011) focused on differences between expert and novice badminton players. Their results showed that there appears to be overlapping regions between experts and novices while observing badminton videos, but it was also supported that novices tend to rely more on visual regions, and experts more on motor regions of the brain. Moreover, Bishop et al. (2013) suggested that high-skill anticipators showed a greater activation of mirror neuron related regions of the brain. Increased brain activity by experts was also supported by Wright et al. (2010), and Milton et al. (2007) proposed that the extensive practice over long time leading to expert performance is reflected by a focused and efficient organization of the neural networks related to a particular task. Thus, physical experience and motor representations appears to be important in order for action observation to be similar to action execution. However, there are still limited knowledge regarding the association between successful action anticipation, expertise, and neural response.

One reason why this is still uncertain may be that most of the studies have used passive control conditions. Hence, less attention has been given to examining what constitutes a successful anticipation, and, if such behavior relies on different neural systems between experts and novices. This is a novel step to analyze action observation by also include the performance of the observer into the analysis. This is important if we want to understand the possibilities of using action observation in practice and in order to provide guidelines in e.g., a clinical setting in which action observation is frequently used (Celnik et al., 2008).

The aim of this pilot study was therefore to examine if an action observation paradigm comparing expert athletes to novices could (1) capture the superior performance of expert athletes and (2) capture the underlying neural mechanisms of successful action observation in relation to skill level. We hypothesized that only the expert athletes could recruit motor regions of the brain and thereby access the motor representations when successfully anticipating actions. Moreover, we hypothesized that novices would recruit visual regions to a greater extent and use cognitive resources of the brain when successfully anticipating actions, which should be reflected by increased activity in pre-frontal cortex.

## MATERIALS AND METHODS

### PARTICIPANTS

For the behavioral part of this pilot, 17 male subjects participated voluntarily. Six of these participants were professional ice hockey players from the Swedish second division (experts, mean age 23.6 ± 4.1 years with 5.33 ± 4.5 years playing on professional level). Five of the participants were from the Swedish fifth division (amateurs, mean age 24.9 ± 2.6 years with 4.25 ± 3.6 years playing on amateur level). Finally, six of the participants had never played ice hockey regularly (novices, mean age 23.4 ± 1.8 years). The novices were students at the university with no hockey or team sport experience; neither did they attend games or sporting events regularly. Three participants from the expert group and three participants from the novice group also participated in the functional magnetic resonance imaging (fMRI) part of this pilot. No amateurs participated in the fMRI. All subjects participated voluntarily, reported right-handedness and were neurologically healthy. They all gave their informed consent and this study followed the ethical standards of the declaration of Helsinki.

### STIMULUS MATERIAL

We decided to use ice hockey as the sport for our stimulus material because it allows one to capture different situations that are likely to appear as real game situations. Also, an extension of the present paradigm would be to look at more complex game situations with several players involved which would make hockey a good candidate in order to create more complex situations based on this initial pilot study. Moreover, there are several local teams available at different skill levels (from professional players to amateurs) making it ideal for us to be able to study skill differences. For the creation of stimulus material one ice hockey player from a Swedish ice hockey high school was used as a shooter and was given instructions how to perform the different shots. The video clips were recorded with a JVC Everio GZ-MG330HAG hard disk camcorder. The video camera was placed behind the goal. The position was chosen in order to be able to see the whole goal, as well as the shooter from the front, to facilitate the prediction of the puck direction. By using such a paradigm we will likely create a situation in which we will be able to compare functional brain response between experts and novices that will reflect differences based on their motor expertise. Since action perception and action execution has been suggested to recruit similar neural regions and also share a common representational domain (Prinz, 1997) we should thereby be able to study motor skill and motor representations.

The same stimulus material was used, and presented in a similar manner, both outside the scanner and during brain imaging. In total, 40 different video clips (10 clips for each location of the goal) of the shooting ice hockey player were used. Adobe Premier Pro CS4 was used to cut all clips into three different time occlusions to reflect the different levels of difficulty. First, all clips were cut when the puck left the stick (easy occlusion). Then, the same video was also cut 300 ms (medium occlusion) and 600 ms (difficult occlusion) before the puck left the stick. Each video clip was about 2 s long depending on which occlusion time that was used. Each video clip was shown three times with different occlusions each time, thus, the total amount of video clips was 120. Original resolution was 720 × 480 pixels (standard PAL video, 4:3 interlaced), but it was cropped to 416 × 480 pixels in order to remove unwanted information in the picture. E-Prime 2.0 was used to randomize the clips and to collect response data.

### PROCEDURE

**FIGURE 1:**
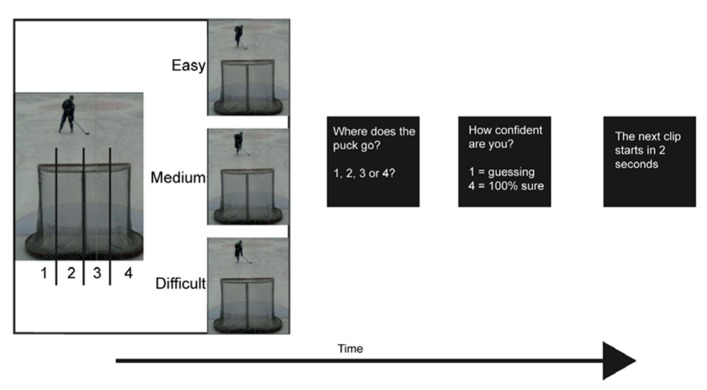
**Experimental setup.** The video clips lasted for about 2 s depending on which occlusion (easy, medium, difficult) that was observed. There was no time limit for the response. The participants viewed a total of 120 video clips.

Before the start of the experiment the participants were given five trials of video clips that were not used in the actual test. The instruction for both the trials and the experiment was to watch each video clip as if they were the player. After each video clip the participants were asked where in the goal the puck would go, they responded by pressing a button. They had four alternatives; they pressed one if they thought that the puck would go to the furthest left, and four if they thought that the puck would go to the furthest right. They could use as much time as they needed to make their response. Then the participants were asked to respond to how confident they were of their answer. Again, they had four alternatives, pressing one if they guessed and four if they were 100% sure of their answer (see **Figure 1**). The same procedure was done in the behavioral part of this pilot as well as during fMRI scanning. During scanning the participants were placed in the scanner and watched a video screen via a tilted mirror on the head coil. They had a response pad under their right hand, attached with Velcro. The participants had earplugs and a headphone to reduce the noise from the scanner and cushions were placed in the coil to minimize head movements. They were also informed not to move any body parts during scanning.

### BRAIN IMAGING PARAMETERS

The fMRI session was conducted on a GE3.0 T system (USA) collecting Blood oxygen level dependent T2* weighted images. The following imaging parameters were used: repetition time 2000 ms; 37 slices with a thickness of 3.4 mm, echo time 30.0 ms, flip angel 80°; field of view 25 cm × 25 cm, matrix 96 × 96. Before statistical analysis pre-processing steps were carried out using SPM5 (Wellcome Department of Cognitive Neurology, London, UK) including: slice timing correction, realignment, unwarping, normalization to an EPI template in the Montreal Neurological Institute space, and finally spatial smoothing (8 mm Gaussian filter). In-house developed software (DataZ) was used for visualization of the results.

### STATISTICAL ANALYSIS

To capture performance the mean value of number of correct answers at each difficulty level (easy, medium and difficult occlusion time) was counted. Subjects’ confidence on their answers was calculated as mean value on each difficult level. A 3 × 3 (3 skill levels × 3 difficulty levels) ANOVA was used to analyze behavioral data between groups. Least significant difference was used as a *Post-Hoc* test. Level of significance was set to *p* < 0.05.

Imaging data was analyzed in respect to observation as well as during decision-making, therefore these two conditions were defined as separate regressors. The observation regressor was based on the entire length of each video clip, whereas the decision regressor was the length up until the participants gave a response. Our main aim was to investigate prediction of the action outcome in relation to successful performance. Since previous studies have not contrasted successful trials vs. unsuccessful trials in their analyzes, we are still uncertain what constitutes successful action anticipation. Relying on passive control conditions, as has been done in prior studies, is a liberal approach in fMRI studies. In the present study the more conservative analytic approach motivates the use of a pilot study before testing the hypotheses in a large-scale attempt. Therefore, single subject contrasts were set up using the general linear model and statistical parametric maps were generated using t-statistics. The main contrast used was comparison of functional brain response during correct vs. incorrect (correct > incorrect) response. This was done for both the observation as well as the decision-making. We then performed a random-effect analysis for each group separately. We used a significant level of *p* < 0.005 uncorrected in the analysis. The voxel-wise threshold was motivated since this study should be considered as a pilot requiring more of a descriptive approach. However, in order to minimize Type I errors, a cluster threshold of minimum five voxels was also applied.

## RESULTS

### BEHAVIORAL DATA

Between group analysis revealed significant difference in performance on easy level (*F*_2,1__4_) = 3.739, *p* < 0.05 and difficult level (*F*_2,1__4_) = 4.653, *p* < 0.05, effect sizes were small to moderate, η^2^ = 0.025 and 0.045 respectively. There was a tendency to significant difference in performance on medium level, *p* = 0.061. *Post Hoc* test showed significant differences between experts and novices on both easy and difficult level *p* < 0.05 and also between amateurs and novices on difficult level *p* < 0.05. Both groups were equally confident in their responses.

### fMRI DATA

Distinct differences in recruited brain regions were revealed between experts and novices both during action observation but also during action decision. The activation pattern for novices during observation (see **Table 1** for exact location in MNI-space, *T*-values, and cluster extent) was mainly associated with regions of the visual cortex, especially at the difficult occlusion. During action decision (**Figure 3**), regions of the pre-frontal cortex, such as middle, superior and inferior frontal cortex was mainly recruited (see **Table 2** for exact location in MNI-space, *T*-values, and cluster extent).

**Table 1:** Brain regions and local maxima of clusters, recruited by novices during action observation of successful trials compared to unsuccessful trials.

Difficulty level	Brain region	*k*	*X*	*Y*	*Z*	*T*
Easy	Temporal pole (BA 38)	5	-52	10	-14	2.8
	Middle temporal gyrus (BA 20)	9	44	8	-34	2.8
Medium	Cerebellum	87	-10	-48	4	10.5
	Fusiform gyrus (BA 37)	87	-28	-38	-20	7.5
	Calcarine (BA 17)	6	-14	-58	10	4.7
Hard	Superior occipital gyrus (BA 19)	46	-18	-82	42	11.9
	Superior occipital gyrus (BA 19)	7	26	-86	36	9.0
	Superior occipital gyrus (BA 19)	7	20	-92	30	6.4
	Precentral gyrus (BA 6)	34	-20	-16	64	6.3
	Middle temporal gyrus (BA 21)	16	-60	-48	-4	5.2
	Cuneus (BA 18)	8	22	-68	20	5.0

*In the analyses a voxel-wise threshold of 0.005 uncorrected was used in combination with a cluster threshold of minimum five voxels.
*

**Table 2:** Brain regions and local maxima of clusters, recruited by novices during action decision of successful trials compared to unsuccessful trials.

Brain region	*k*	*X*	*Y*	*Z*	*T*
Middle frontal cortex (BA 8)	251	32	18	56	55.2
Inferior temporal sulcus (BA 48)	128	-38	22	16	42.3
Caudate	79	-6	12	6	26.0
Lingual gyrus (BA 19)	14	-22	-68	0	21.5
Inferior temporal sulcus (BA 48)	42	30	30	26	18.0
Insula (BA 47)	48	-30	22	-2	18.0
Superior medial frontal cortex (BA 10)	53	4	34	52	15.5
Inferior frontal gyrus (BA 45)	49	46	32	16	14.4
Caudate	18	8	16	6	14.1
Middle frontal cortex	38	32	28	38	13.6
Rolandic operculum (BA 6)	13	-46	4	16	11.8

*In the analyses a voxel-wise threshold of 0.005 uncorrected was used in combination with a cluster threshold of minimum five voxels.*

For experts, the activation pattern during action observation involved mainly regions in the superior and middle temporal gyri, but also the pre-motor cortex. Interestingly, these regions were again recruited during action decision (see **Tables 3** and **4** for exact location in MNI-space, *T*-values, and cluster extent). Thus, novices recruited more visual and frontal regions and experts more motor and temporal regions during successful action anticipation.

**Table 3:** Brain regions and local maxima of clusters, recruited by experts during action observation of successful trials compared to unsuccessful trials.

Difficulty level	Brain region	*k*	*X*	*Y*	*Z*	*T*
Easy	Precentral gyrus (BA 6)	30	36	-26	68	9.8
	Middle frontal gyrus (BA 8)	5	-28	36	44	5.8
	Supplementary motor area (BA 6)	13	10	2	76	4.2
Medium	Cerebellum	30	14	-50	-40	8.5
	Cerebellum	50	38	-56	-38	8.2
	Superior temporal gyrus (BA 42)	50	60	-38	18	7.4
	Precentral gyrus (BA 6)	7	62	4	30	7.0
	Middle temporal gyrus (BA 21)	14	-50	-44	6	5.5
Hard	Middle temporal gyrus (BA 21)	55	-52	4	-32	9.3
	Superior frontal cortex (BA 10)	8	-10	58	8	7.6
	Temporal pole (BA 38)	55	-48	14	-28	6.2
	Middle temporal gyrus (BA 21)	23	-48	-40	4	5.4
	Middle temporal gyrus (BA 21)	10	-44	-6	-14	5.0

*In the analyzes a voxel-wise threshold of 0.005 uncorrected was used in combination with a cluster threshold of minimum five voxels.
*

**Table 4:** Brain regions and local maxima of clusters, recruited by experts during action decision of successful trials compared to unsuccessful trials.

Brain region	*k*	*X*	*Y*	*Z*	*T*
Middle temporal gyrus (BA 21)	17	-56	-28	-2	11.0
Post central gyrus (BA 4)	8	20	-34	78	10.7
Pre-motor cortex (BA 6)	10	-6	12	72	10.1
Superior temporal gurys (BA 41)	15	50	-34	14	8.0
Inferior temporal gyrus (BA 37)	14	-56	-64	-10	7.0
Superior temporal gyrus (BA 21)	16	64	-28	8	5.8
Middle temporal gyrus (BA 21)	13	-52	6	-30	5.7

*In the analyses a voxel-wise threshold of 0.005 uncorrected was used in combination with a cluster threshold of minimum five voxels.*

**FIGURE 2:**
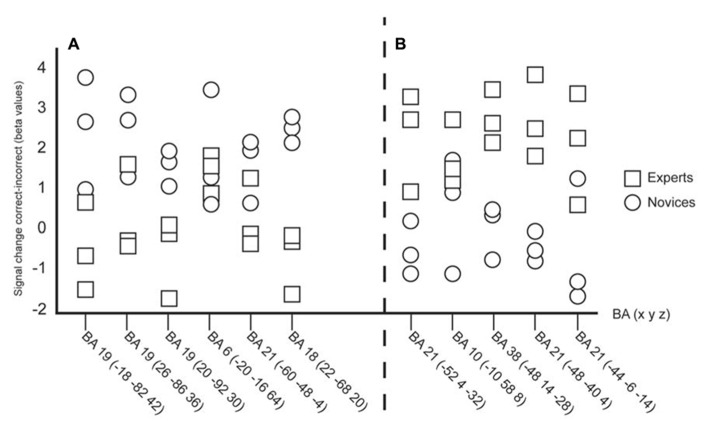
**(A)** Individual plots of the local maxima ) during observation of the early occluded clips. **(B)** Individual plots of the local maxima revealed from the clusters by the experts (see **Table 3**) during observation of the early occluded clips. The individual plots confirm that experts and novices are using separate neural networks during successful anticipation of actions.

Based on the local maxima revealed from the early, difficult, occluded video clips individual data were plotted (**Figure 2**) in order to examine individual variability. There were six peaks from the novices and five peaks from the experts plotted (see **Tables 1** and **3**). The individual plots reveal that even though there are some overlap between experts and novices, the overall results show that distinct neural networks are used depending on level of expertise.

**FIGURE 3:**
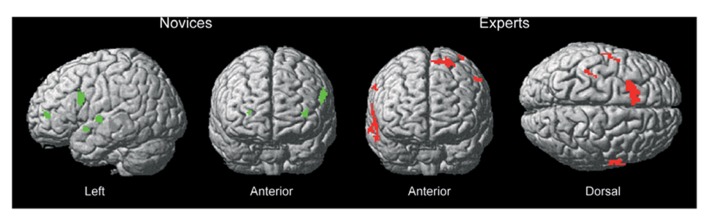
**Brain regions recruited for novices and experts during action decision of successful trials compared to unsuccessful trials.** Novices rely on pre-frontal regions whereas experts rely on motor/pre-motor regions of the brain. The functional brain response is overlaid on a rendered standardized brain (MNI-space) with a threshold of 0.005 uncorrected only showing clusters with a minimum of five voxels.

## DISCUSSION

In the present pilot study we investigated the association between successful action anticipation, expertise, and underlying neural mechanisms. The results are promising and indicate that the present paradigm is suitable to use when studying how motor experience affects our ability to understand relevant information and make a correct anticipation during action observation. However, since the presented data is only from a pilot study great caution should be undertaken when interpreting the results, and before generalizing the results, a large-scale attempt should confirm the findings. The present study followed the logic and stages suggested by Williams and Ericsson (2005) as an approach to study perceptual-cognitive relations in respect to action observation. The first stage was to capture the expert performance. The behavioral data showed that the paradigm was able to separate performance between different groups of individuals with different amount of hockey skill. This is important because it strengthens the validity of the paradigm. Thus, the results further underpin prior evidence regarding how experts outperform novices in action anticipation (Abernethy and Zawi, 2007; Aglioti et al., 2008). We only found a tendency of significant performance differences between experts and amateurs in the behavioral part of this pilot. This is also similar to what others have reported (Wright et al., 2011), which is likely an indication about how difficult it is to create a paradigm that is complex enough to separate the behavior between high skill level and intermediate skill level, but at the same time not too difficult for novices. It would have been interesting to also have fMRI data on the amateurs since that would give us an indication regarding how much experience that is necessary to recruit similar brain regions as experts. A recent study suggested that intermediate skilled level badminton players recruit brain regions more similar to novice players than expert players during anticipation tasks, although the anticipation performance of the intermediate skilled players was better than the novices (Bishop et al., 2013). The second stage, according to Williams and Ericsson (2005), is to identify the underlying mechanisms. In the present study, we focused on the underlying *neural* mechanisms. The results showed that even the functional brain response during action observation and action decision differ between experts and novices when comparing successful trials with unsuccessful trials. Williams and Ericsson also emphasized on a third stage, to examine how expertise is developed. That stage was not covered in the present study. It has been suggested that most research has focused on capturing expert performance (Williams and Ericsson, 2005), thus the present study has the potential to also deepen our understanding regarding mechanisms associated to experts’ superiority during action observation.

The main aim for the fMRI data was to compare correct versus incorrect trials. Thus, this was an attempt to clarify the functional brain regions required for a successful anticipation of actions, and whether such regions differ between novices and experts. Potential differences in underlying neural mechanisms will give us information about if a successful anticipation is handled similar depending on level of motor skill, or if motor experience alters the functional response of the brain. This is a different, and more conservative, approach compared to most previous studies. Interestingly, a similar approach was done recently when Abreu et al. (2012) investigated accuracy in anticipation. They did not, however, make a comparison of successful vs. unsuccessful trials as the present paper, yet, their results confirmed that novices appears to use more pre-frontally oriented brain regions, compared to experts that have increased activity in insular cortex.

Our results indicate that experts, when successfully anticipated action outcome, primarily relied on motor regions in combination with regions of the temporal lobe. Novices instead relied on the visual system during action observation, which is similar to what has been noted during low-level of visual processing (Takahashi et al., 2008). Hence, it appears as if the novices had to search for valuable information in the video clips. Then, during action decision, novices relied on pre-frontal cortex to decide the fate of the action. Pre-frontal cortex is often associated with cognitive demanding tasks such as memory and executive functions (Cabeza and Nyberg, 2000). Thus, it appears as if they needed to use cognitive resources to solve the problem regarding the fate of the action. Experts on the other hand recruited motor regions, which is interpreted as if the experts were able to recruit regions where complex motor representations are stored (Meister et al., 2005), these regions are also well known mirror neuron regions (Rizolatti and Craighero, 2004). By recruiting these regions the experts gathered information from the motor representations, which comprised the result of the action (see Kandel et al., 2000), and thereby could decide the fate of the action. Interestingly, previous results have shown that experts use fewer eye fixations compared with novices when observing actions (Mann et al., 2007), experts are able to extract more meaningful information in shorter time (Williams et al., 1999), and experts have developed specific perceptual-cognitive mechanism to better and more effectively read advanced body cues (Williams et al., 2002). Thus, we suggest that a possible explanation for such behavior may be because experts do not have to rely on a visual search strategy, instead they can directly extract the information from the motor representations and analyze the interaction much faster using parts of the temporal lobe. Activity in the temporal lobe has previously been suggested to reflect analysis of complex human body movements (e.g., dancing) based on kinematic cues in which valuable information is extracted in order to interact with others (Allison et al., 2000). Cross et al. (2006) interpreted activity in the temporal regions as reflecting increased visual scene processing demands. Our study does not offer full support for such conclusion since we contrasted correct and incorrect answers in the analysis. Thus, activation in temporal regions during action observation is not interpreted to reflect visual processing demands since such demand was equal in the two conditions. Cerebellum was also recruited probably reflecting its involvement in movement production and, it has even been proposed that Cerebellum may be interconnected with the mirror neuron system (Sokolov et al., 2010). Taken together, the present pilot study suggests that level of motor skill affects the functional brain response during successful action anticipation.

In the present paper, based on the contrasts performed, we did not find any parietal activation. Parietal activity has been frequently associated with action observation in general (e.g., Buccino et al., 2001), as well as associated with expert athletic performance (see also Yarrow et al., 2009). In a study specifically targeting the role of parietal cortex in prediction of incoming motor actions, Fontana et al. (2012) measured EEG activity in patients with lesions in the parietal lobe while watching a video of a person grasping an object. Results showed how individuals with intact parietal lobe (healthy controls) experienced a readiness potential within the parietal lobe preceding the observation of the upcoming action. No such potential was present in the parietal patient group. Moreover, parietal activation has also been shown by experts more so than novices in studies of motor planning, possibly reflecting their ability for global rather than selective attention, suggesting that experts are more focused and have a more efficient organization (Milton et al., 2007). Thus, the parietal lobe is most likely involved in the prediction of incoming motor actions, which the present study do not argue against. However, based on this data we do not offer support that the parietal cortex is involved when separating between correctly or incorrectly anticipated actions.

One critical point of novelty in the present study is the use of unsuccessful trials as baseline. By doing so we isolated the functional brain response associated with successful anticipation, which in turn is associated with skill. Thus, the differences in brain regions between the groups of novices and experts are based on their hockey skills and not their ability to make correct anticipations. This highlights that even though there are similarities between experts and novices during action anticipation (see e.g., Abreu et al., 2012), when investigating successful anticipation, experts and novices tend to rely on partly different neuronal networks, with experts relying more on motor representations and medial temporal lobe, and novices more on visual and pre-frontal regions. Interestingly it has been suggested that anticipatory information pick-up is related to highly domain-specific memory structures (Yarrow et al., 2009) implying that motor representation created by physical training (in the present paper hockey training) in combination with medial temporal lobe, which is a structure highly involved in memory, is a plausible explanation to our findings. The novices do not have the memories (motor representations) for the actions, and thus must rely on different strategies to successfully perform the task, which was reflected by the use of altered neural systems.

Obviously, because of the limited sample of participants, one must interpret the results from this pilot with great caution, and the results should be confirmed with more participants before generalizing. However, it highlights some interesting findings for future studies of this topic. We must, however, perform this study in large scale with more participants to fully appreciate the proposed relationships. Further, in the present study, even though we used a complex task, it was only with one player. Adding players to create a more complex scenario would be interesting and probably more demanding on the system. However, it is likely that key brain regions for such task will be revealed within similar regions as in the present study. In conclusion, data from the present study support that depending on the level of motor skill of the observer (i.e., task specific physical experience), when correctly anticipating actions different neural systems will be recruited.

## Conflict of Interest Statement

The authors declare that the research was conducted in the absence of any commercial or financial relationships that could be construed as a potential conflict of interest.
